# Trends in prevalence, mortality, and disability-adjusted life-years relating to chronic obstructive pulmonary disease in Europe: an observational study of the global burden of disease database, 2001–2019

**DOI:** 10.1186/s12890-022-02074-z

**Published:** 2022-07-28

**Authors:** Dominic C. Marshall, Omar Al Omari, Richard Goodall, Joseph Shalhoub, Ian M. Adcock, Kian Fan Chung, Justin D. Salciccioli

**Affiliations:** 1 National Heart and Lung Institute, Imperial College London, London, UK; 2 Medical Data Research Collaborative, London, UK; 3 Mount Auburn Hospital, Cambridge, MA USA; 4 Harvard Medical School, Boston, MA USA; 5 Oxford University Hospitals NHS Foundation Trust, Oxford, UK; 6 Imperial College Healthcare NHS Trust, London, UK; 7 Department of Surgery and Cancer, Imperial College London, London, UK; 8 Royal Brompton Hospital, London, SW3 UK; 9 Division of Pulmonary and Critical Care Medicine, Brigham and Women’s Hospital, Boston, MA USA

**Keywords:** Chronic obstructive pulmonary disease, COPD, Mortality, Prevalence, Trends

## Abstract

**Introduction:**

Chronic Obstructive Pulmonary Disease (COPD) is associated with significant mortality and well-defined aetiological factors. Previous reports indicate that mortality from COPD is falling worldwide. This study aims to assess the burden of COPD using prevalence, mortality, and disability-adjusted life years (DALYs) between 2001 and 2019 in 28 European countries (the European Union and the United Kingdom).

**Methods:**

We extracted COPD data from the Global Burden of Disease database based on the International Classification of Diseases versions 10 (J41, 42, 43, 44 and 47). Age-standardised prevalence rates (ASPRs), age-standardised mortality rates (ASMRs), and DALYs were analysed for European countries by sex for each year (2001–2019) and reported per 100,000 population. We used Joinpoint regression analysis to quantify changing trends in the burden of COPD.

**Results:**

In 2019, the median ASPR across Europe was 3230/100,000 for males and 2202/100,000 for females. Between 2001 and 2019, the median percentage change in ASPR was − 9.7% for males and 4.3% for females. 23/28 countries demonstrated a decrease in ASPRs in males, and 11/28 demonstrated a decrease in females. The median percentage change in ASMR between 2001 and 2019 was − 27.5% for males and − 10.4% for females. 25/28 and 19/28 countries demonstrated a decrease in ASMR in males and females, respectively.

**Conclusion:**

In the EU between 2001 and 2019 COPD prevalence has overall increased in females but continues to decrease in males and in some countries, female prevalence now exceeds that of males. COPD mortality in the EU has decreased overall between 2001 and 2019; however, this decrease is not universal, particularly in females, and therefore remains a substantial source of amenable mortality.

**Supplementary Information:**

The online version contains supplementary material available at 10.1186/s12890-022-02074-z.

## Introduction

In 2015, Chronic Obstructive Pulmonary Disease (COPD) had an estimated prevalence of 174.5 million people with approximately 3.2 million deaths globally [1]. Although mortality across Europe is falling, COPD remains a significant burden and financial cost to healthcare systems [2]. Effective public health interventions targeting aetiological factors, such as smoking and air pollution, are important for reducing the ongoing burden of disease.

While analyses of the Global Burden of Disease (GBD) and the Eurostat databases indicate that overall mortality from COPD is falling (particularly in males), this decrease is not uniform across Europe [2–4]. Although public health initiatives and social change have resulted in decreased smoking rates, there is still an unacceptably high uptake of smoking in younger generations where there should be room for public health interventions [5]. Furthermore, high levels of air pollution, particularly in cities and an ageing population, may contribute to a flattening of the previously observed falling mortality.

A recent update has reported on the burden of respiratory disease across the globe, but there have been no recent reports focusing on changing trends within Europe [4]. Therefore, our primary objectives were to compare the trends in the prevalence and mortality from COPD across Europe (the European Union and the United Kingdom); to evaluate the differential burden of disease using disability-adjusted life-years (DALYs) and to provide an indicator of population-based case-fatality using mortality-incidence ratios. To achieve this, we used the GBD database for the European Union (EU) member states (including the United Kingdom) over the period 2001–2019 to determine the prevalence, mortality, mortality-incidence ratios and DALYs. We also describe the changing trends in the burden of COPD using Joinpoint regression analysis.

## Methods

### Data source

Data for COPD incidence, mortality and DALYs were extracted from the GBD database from 2001 to 2019 for European Union (EU) member states and the United Kingdom (UK). Briefly, this is an extensive, publicly available database compiled from data sets from 127 countries that report mortality and disability data (deaths, death rates, years of life lost due to premature mortality, prevalence, and incidence) as commissioned by the World Health Organisation (WHO). The precise GBD methodology is described elsewhere [6–8], and we have previously used similar methodology in observational analyses of trends in the disease burden of type two diabetes mellitus [9], peripheral arterial disease [10], abdominal aortic aneurysm [11], and lower extremity amputation [12]. The GBD uses systematic reviews, survey data, disease registries, hospital administrative data, claims, inpatient and outpatient data, and case notifications as data sources to estimate disease incidence. Classification of disease is based on the International Classification of Disease (ICD) coding system 10th revisions using J41, J42, J43, J44 and J47 for COPD.

COPD incidence was computed along with prevalence using detailed methods reported elsewhere [13]. Briefly, cases were defined as per the Global Initiative for COPD (GOLD) definition of COPD as a ratio of < 0.7 of the FEV1/FVC measured by spirometry after bronchodilation. Data were obtained from a recent meta-analysis [14] in addition to survey data with spirometry measurements. Data using alternative case-definitions with age-specific ratios were cross-walked from studies reporting prevalence with alternative case definitions. A three-step modelling strategy was used: first, the incidence was estimated using the DisMod-MR 2.1 model, followed by separate estimation of the proportions by GOLD severity of disease in DisMod-MR 2.1, and then finally, a combination of these processes to derive prevalence by severity. Population estimates and confidence intervals are then produced using Bayesian statistical methods [13].

For COPD mortality, estimates were computed using the GBD Cause of Death Ensemble model (CODEm). Data for the model were obtained from vital registration and surveillance data from the cause of death database. Outlier criteria were used to exclude data points that were implausibly high/low, substantially conflicted with established age or temporal patterns, or substantially conflicted with other data sources from the same or similar locations [8].

DALYs were calculated by the GBD group as the sum of years of life lost (YLL) and years lived with disability (YLD). GBD methodology computes YLL by multiplying the standard life expectancy at the age of death by the estimated number of deaths (per cause) [13]. YLD were calculated by multiplying the prevalence of COPD (estimated using the DisMod MR 2.1 tool as described earlier) by the disability-weighting for COPD (gathered from the GBD 2013 European disability weights measurement study [7]. YLDs were then corrected for co-morbidities via a microsimulation process before combining with YLLs to give estimated DALYs and their uncertainties.

### Handling of the GBD data

Country-specific age-standardised prevalence rates (ASPRs), age-standardised death rates (ASMRs) and DALYs per 100,000 population by sex and year for COPD were extracted from the GBD Results Tool for each year between 2001 and 2019, inclusive, for each EU member state. Data in the GBD database are age-standardised through computing a standard population using a non-weighted average across a percentage of the population of all countries in each five-year age bracket (years 2010–2035) from the United Nations Population Division’s World Population Prospects (2012 revision).

Absolute and relative changes in ASPRs and ASMRs over the observation period (i.e., differences between the rates in 2001 and 2019) were calculated by each sex for each country. ASMRs were quantified as a proportion of age-standardised incidence rates (ASIR) by dividing ASMR by the ASIR to calculate a mortality-to-incidence index (MII) for each year (2001–2019, inclusive) for each sex in each country. MII provides an estimate as to case fatality for COPD but is not corrected for the lag in the natural history of the disease.

The GBD quantifies the availability and completeness of the mortality data by each location-year to indicate the reliability of cause of death data. Each country is graded on a 5-star scale [8]. For the countries analysed in the present analysis, except for Cyprus and Slovakia (2-stars and 3-stars, respectively), 15 EU countries scored 4-stars (Belgium, Bulgaria, Croatia, Czech Republic, Denmark, France, Germany, Greece, Luxembourg, Netherlands, Poland, Portugal, Romania, Slovenia, and Spain), representing greater than 65% completeness of mortality data. The 11 remaining EU countries have 5-star data, demonstrating greater than 85% completeness of the data (Austria, Estonia, Finland, Hungary, Ireland, Italy, Latvia, Lithuania, Malta, Sweden, and the UK).

### Statistical analysis

Joinpoint regression analysis was used to assess trends in the disease burden of COPD. The Joinpoint software (Joinpoint Command Line Version 4.5.0.1) was developed and provided by the United States National Cancer Institute Surveillance Research Program [15]. This software models trends in data over time then fits the simplest model possible to the data by connecting several different line segments on a logarithmic scale. These segments are known as ‘Joinpoints’, with the simplest model (i.e., 0 Joinpoints) being an uninterrupted line. Sequentially Joinpoints are added and each new model is tested for significance using a Monte Carlo permutation method. The software also gives estimated annual percentage changes (EAPC) for each line segment. Each EAPC is tested to establish if a difference exists from the null hypothesis of no change. Consequently, the final model consists of zero or multiple Joinpoints, where Joinpoints are added each represents a statistically significant (*p-*value < 0.05) change in trend (increase or decrease), with each trend described by the EAPC and the associated confidence intervals. The EAPC allows the assessment of trend changes at a constant percentage per year.

## Results

We included 28 countries in the final analysis with 19 years of data, with no missing data or need for imputation. Over the 19-years period, changes were observed in COPD mortality, prevalence and DALYs across the 28 European countries. Countries with the three highest and three lowest rates for each metric are reported in descending and ascending order respectively.

### Prevalence

Table 1 demonstrates ASPR per 100,000 per country in 2001 and 2019. In 2019, the median ASPR across Europe was 3230/100,000 and 2202/100,000 for males and females, respectively. For males, the highest ASPRs in 2019 were observed in Belgium, Denmark, and Hungary (4345/100,000, 4214/100,000 and 4123/100,000, respectively). For females, the highest ASPRs in 2019 were observed in Denmark, the Netherlands, and the UK (4378/100,000, 3990/100,000, and 3900/100,000, respectively). The lowest ASPRs in 2019 in males were observed in Latvia, Estonia, and France (1844/100,000, 1936/100,000, and 2351/100,000). For females, the lowest ASPRs were observed in Latvia, Lithuania, and Estonia (970/100,000, 1061/100,000, and 1082/100,000, respectively). Between 2001 and 2019, the median change in ASPR for males was − 372.9/100,000, with a median percentage change of − 9.7%. For females, the median change in ASPR was 97/100,000, with a median percentage change of 4.3%. The greatest reduction in ASPR between 2001 and 2019 was seen in Lithuania for both males (− 32.1%) and females (− 29.9%). 23 of the 28 countries demonstrated decreased ASPRs in males, while only 11 countries demonstrated decreases in females.

**Table 1:** 2001and 2019 values for age-standardised prevalence rate (ASPR) per 100,000, age-standardised death rate (ASMR) per 100,000, mortality-to-incidence ratio (MII) and disability adjusted life year (DALYs) per 100,000 for COPD

	Prevalence (ASPR per 100,000)			Mortality (ASDR per 100,000)			Mortality Incidence Indices			DALYs per 100,000		
Country	2001	2019	Change (%)	2001	2019	Change (%)	2001	2019	Change (%)	2001	2019	Change (%)
*Female*												
Austria	2679	2816	137.16 (5.1)	10.78	10.89	0.11 (1.0)	0.06	0.06	0 (− 2.0)	286	303	17.09 (6.0)
Belgium	3208	3588	380.58 (11.9)	18.19	16.81	− 1.38 (− 7.6)	0.08	0.07	− 0.01 (− 15.4)	472	487	14.97 (3.2)
Bulgaria	1961	2318	357.2 (18.2)	17.22	12.28	− 4.94 (− 28.7)	0.15	0.09	− 0.06 (− 39.9)	399	346	− 53.68 (− 13.4)
Croatia	1858	2132	274.08 (14.8)	10.02	11.28	1.26 (12.6)	0.08	0.07	− 0.01 (− 11.0)	272	290	17.72 (6.5)
Cyprus	2322	2266	− 55.91 (− 2.4)	24.5	20.06	− 4.44 (− 18.1)	0.14	0.11	− 0.02 (− 16.3)	442	357	− 85.54 (− 19.3)
Czech Republic	1730	2141	410.63 (23.7)	8.15	10.56	2.41 (29.6)	0.08	0.08	0 (− 2.5)	248	307	59.44 (24.0)
Denmark	4235	4378	143.11 (3.4)	36.12	30.07	− 6.05 (− 16.8)	0.13	0.10	− 0.03 (− 22.5)	910	742	− 168.49 (− 18.5)
Estonia	995	1082	86.19 (8.7)	5.4	4.85	− 0.55 (− 10.1)	0.11	0.08	− 0.02 (− 23.7)	154	147	− 7.43 (− 4.8)
Finland	1590	1697	107.78 (6.8)	6.41	6.45	0.04 (0.6)	0.07	0.06	− 0.01 (− 10.2)	206	210	3.45 (1.7)
France	1734	1650	− 83.79 (− 4.8)	8.31	6.48	− 1.83 (− 22.1)	0.06	0.05	− 0.01 (− 12.9)	170	162	− 8.22 (− 4.8)
Germany	2754	3213	459.04 (16.7)	11.01	13.37	2.36 (21.4)	0.06	0.06	0 (5.5)	367	433	65.12 (17.7)
Greece	2288	2547	258.89 (11.3)	13.98	15.00	1.02 (7.3)	0.08	0.08	0 (− 1.1)	348	384	35.67 (10.3)
Hungary	2727	3097	369.22 (13.5)	15.33	19.86	4.53 (29.6)	0.1	0.11	0.01 (12.3)	447	569	121.49 (27.2)
Ireland	4103	3687	− 416.52 (− 10.2)	30.93	23.03	− 7.9 (− 25.5)	0.11	0.09	− 0.02 (− 18.7)	698	541	− 156.6 (− 22.4)
Italy	1936	1878	− 57.93 (− 3.9)	10.02	9.46	− 0.56 (− 5.6)	0.07	0.06	0 (− 3.6)	248	233	− 15.51 (− 6.2)
Latvia	1011	970	− 40.94 (− 4.1)	5.46	5.06	− 0.4 (− 7.3)	0.10	0.09	− 0.01 (− 11.1)	154	140	− 14.02 (− 9.1)
Lithuania	1513	1061	− 452.56 (− 29.9)	8.92	5.67	− 3.25 (− 36.4)	0.11	0.09	− 0.01 (− 11.2)	249	159	− 89.55 (− 36.0)
Luxembourg	3083	3290	207.04 (6.7)	14.67	13.10	− 1.57 (− 10.7)	0.07	0.05	− 0.01 (− 17.6)	409	390	− 19.34 (− 4.7)
Malta	1781	1731	− 50.02 (− 2.8)	6.17	4.50	− 1.67 (− 27.1)	0.05	0.04	− 0.01 (− 23.8)	199	169	− 30.13 (− 15.1)
Netherlands	3545	3990	444.85 (12.6)	21.43	20.91	− 0.52 (− 2.4)	0.09	0.08	− 0.01 (− 14.8)	575	584	9.33 (1.6)
Poland	1789	1794	4.61 (0.3)	8.97	7.95	− 1.02 (− 11.4)	0.09	0.07	− 0.01 (− 15.5)	277	264	− 13.07 (− 4.7)
Portugal	2456	2119	− 337.55 (− 13.7)	18.98	13.92	− 5.06 (− 26.7)	0.10	0.08	− 0.02 (− 20.1)	417	304	− 112.87 (− 27.1)
Romania	1850	1575	− 274.31 (− 14.8)	18.31	10.28	− 8.03 (− 43.9)	0.14	0.10	− 0.04 (− 27.0)	384	262	− 122 (− 31.8)
Slovakia	1286	1485	199.62 (15.5)	7.1	6.32	− 0.78 (− 11.0)	0.10	0.07	− 0.03 (− 25.6)	200	200	− 0.14 (− 0.1)
Slovenia	1647	1510	− 137.15 (− 8.3)	11.18	6.29	− 4.89 (− 43.7)	0.10	0.06	− 0.04 (− 40.6)	264	182	− 81.72 (− 31.0)
Spain	2433	2263	− 170.08 (− 7.0)	16.68	13.42	− 3.26 (− 19.5)	0.09	0.08	− 0.01 (− 13.1)	321	277	− 43.21 (− 13.5)
Sweden	3160	3371	210.95 (6.7)	11.97	14.10	2.13 (17.8)	0.06	0.06	0 (2.6)	447	451	3.95 (0.9)
United Kingdom	3817	3900	83.54 (2.2)	25.71	26.27	0.56 (2.2)	0.10	0.10	0 (− 1.1)	623	622	− 0.9 (− 0.1)

Country	Prevalence (ASPR per 100,000)			Mortality (ASDR per 100,000)			Mortality incidence indices			DALYs per 100,000		
	Start	End	Change (%)	Start	End	Change (%)	Start	End	Change (%)	Start	End	Change (%)
*Male*												
Austria	3937	3485	− 451.99 (− 11.5)	26.93	21.70	− 5.23 (− 19.4)	0.1	0.09	− 0.01 (− 8.9)	610	520	− 89.78 (− 14.7)
Belgium	4401	4345	− 55.38 (− 1.3)	57.17	36.51	− 20.66 (− 36.1)	0.18	0.12	− 0.06 (− 34.1)	1111	798	− 312.88 (− 28.2)
Bulgaria	3146	3089	− 57.61 (− 1.8)	39.7	29.74	− 9.96 (− 25.1)	0.19	0.14	− 0.05 (− 26.3)	901	711	− 189.49 (− 21.0)
Croatia	3361	3413	52.06 (1.6)	33.32	30.50	− 2.82 (− 8.5)	0.13	0.12	− 0.02 (− 12.7)	726	646	− 80.26 (− 11.1)
Cyprus	3375	3395	19.87 (0.6)	48.64	34.61	− 14.03 (− 28.8)	0.19	0.13	− 0.05 (− 28.6)	799	620	− 179.71 (− 22.5)
Czech Republic	3177	3160	− 17.93 (− 0.6)	21.29	24.76	3.47 (16.3)	0.11	0.11	0.01 (8.7)	578	618	39.77 (6.9)
Denmark	4323	4214	− 109.1 (− 2.5)	51.6	38.80	− 12.8 (− 24.8)	0.17	0.13	− 0.04 (− 23.1)	1065	818	− 247.71 (− 23.3)
Estonia	2091	1936	− 154.54 (− 7.4)	20.85	15.79	− 5.06 (− 24.3)	0.16	0.11	− 0.04 (− 27.6)	460	344	− 116.72 (− 25.4)
Finland	3471	2838	− 632.9 (− 18.2)	27.09	18.65	− 8.44 (− 31.2)	0.11	0.09	− 0.02 (− 17.5)	621	443	− 177.85 (− 28.6)
France	2940	2351	− 589.43 (− 20.1)	22.89	15.00	− 7.89 (− 34.5)	0.11	0.09	− 0.02 (− 16.6)	425	315	− 109.93 (− 25.9)
Germany	3965	3684	− 281.29 (− 7.1)	27.88	25.01	− 2.87 (− 10.3)	0.10	0.10	0 (− 1.8)	689	626	− 63.02 (− 9.2)
Greece	2858	3137	278.82 (9.8)	21.85	23.56	1.71 (7.8)	0.10	0.10	0 (− 0.2)	511	565	53.71 (10.5)
Hungary	4029	4123	94.11 (2.3)	37.88	40.85	2.97 (7.8)	0.13	0.14	0.01 (5.6)	947	1009	61.56 (6.5)
Ireland	4248	3624	− 623.98 (− 14.7)	58.3	34.40	− 23.9 (− 41.0)	0.19	0.13	− 0.06 (− 32.9)	1047	655	− 392.79 (− 37.5)
Italy	3422	2794	− 628.2 (− 18.4)	31.78	23.48	− 8.3 (− 26.1)	0.13	0.11	− 0.02 (− 13.9)	618	453	− 165.66 (− 26.8)
Latvia	2233	1844	− 389 (− 17.4)	19.01	15.08	− 3.93 (− 20.7)	0.14	0.12	− 0.02 (− 11.7)	439	343	− 95.3 (− 21.7)
Lithuania	3495	2373	− 1121.39 (− 32.1)	41.68	21.75	− 19.93 (− 47.8)	0.18	0.13	− 0.05 (− 27.4)	890	476	− 414.82 (− 46.6)
Luxembourg	4286	3438	− 847.99 (− 19.8)	39.24	24.02	− 15.22 (− 38.8)	0.13	0.1	− 0.03 (− 26.5)	843	543	− 299.94 (− 35.6)
Malta	3691	3067	− 624.17 (− 16.9)	35.46	22.75	− 12.71 (− 35.8)	0.13	0.1	− 0.03 (− 24.9)	734	498	− 236.49 (− 32.2)
Netherlands	4178	3743	− 434.71 (− 10.4)	55.89	35.42	− 20.47 (− 36.6)	0.18	0.13	− 0.06 (− 30.8)	1107	740	− 367.71 (− 33.2)
Poland	3390	2873	− 516.38 (− 15.2)	35.37	23.66	− 11.71 (− 33.1)	0.15	0.11	− 0.04 (− 26.5)	806	565	− 241.14 (− 29.9)
Portugal	4101	3396	− 705.02 (− 17.2)	44.69	30.57	− 14.12 (− 31.6)	0.15	0.12	− 0.03 (− 20.8)	902	596	− 305.96 (− 33.9)
Romania	3625	3299	− 325.78 (− 9.0)	49.31	30.43	− 18.88 (− 38.3)	0.20	0.14	− 0.06 (− 28.4)	1095	761	− 334.15 (− 30.5)
Slovakia	2368	2465	96.18 (4.1)	22.92	19.00	− 3.92 (− 17.1)	0.15	0.11	− 0.04 (− 25.3)	547	463	− 83.8 (− 15.3)
Slovenia	3591	2472	− 1119 (− 31.2)	43.98	20.01	− 23.97 (− 54.5)	0.16	0.1	− 0.06 (− 38.9)	872	420	− 451.14 (− 51.8)
Spain	4408	4051	− 356.7 (− 8.1)	60.01	41.61	− 18.4 (− 30.7)	0.19	0.14	− 0.05 (− 26.2)	1046	755	− 291.55 (− 27.9)
Sweden	3447	3055	− 391.6 (− 11.4)	19.69	16.31	− 3.38 (− 17.2)	0.08	0.07	− 0.01 (− 10.4)	465	387	− 78.04 (− 16.8)
United Kingdom	4018	3903	− 114.08 (− 2.8)	44.94	36.91	− 8.03 (− 17.9)	0.16	0.13	− 0.02 (− 15.1)	947	788	− 158.7 (− 16.8)

Change as absolute values and percentages in parentheses reported between 2001 and 2019

### Mortality

Table 1 demonstrates ASMRs per 100,000 per country in both 2001 and 2019 for males and females, respectively. In 2019, the median ASMR across Europe was 24/100,000 and 12/100,000 for males and females, respectively. For males, the highest ASMRs in 2019 were observed in Spain, Hungary, and Denmark (42/100,000, 41/100,000 and 39/100,000, respectively). For females, the highest ASMRs in 2019 were observed in Denmark, the UK, and Ireland (30/100,000, 26/100,000 and 23/100,000, respectively). The lowest ASMRs in 2019 in males were observed in France, Latvia, and Estonia (15/100,000, 15/100,000, and 16/100,000, respectively). For females, the lowest ASMRs in 2019 were observed in Malta, Estonia, and Latvia (5/100,000, 5/100,000 and 5/100,000, respectively). Between 2001 and 2019, the median change in ASMR for males was − 9/100,000, with a median percentage change of − 27.5%. For females, the median change in ASMR was − 1/100,000, with a median percentage change of − 10.4%. The greatest reduction in COPD ASMR between 2001 and 2019 was observed in Slovenia for males (− 54.5%) and Romania for females (− 43.9%). Over the observation period, 25 of 28 countries demonstrated a decrease in ASMR in males and 19 of 28 countries in females.

### Mortality-to-incidence index

Table 1 shows sex-specific MIIs by country. In 2019, median MIIs across Europe were 0.12 for males and 0.08 for females. For males, Spain, Romania, Hungary, and Bulgaria had the highest MII in 2019 (0.14 for all four countries). For females, the highest MIIs in 2019 were observed in Hungary and Cyprus (0.11 in both countries). The lowest MIIs in 2019 in males were observed in Sweden, Austria, Finland, and France (0.07, 0.09, 0.09, and 0.09, respectively). For females, the lowest MIIs in 2019 were observed in Malta, France, and Luxembourg (0.04, 0.05, and 0.05, respectively). Between 2001 and 2019, the median percentage change in MIIs was − 24.0% and − 14.0% for males and females, respectively. 26 of 28 countries demonstrated a decrease in MII in males and 25 of 28 countries in females.

### DALYs

Table 1 shows DALYs per 100,000 per country. In 2019, median DALYs per 100,000 across Europe were 581/100,000 and 304/100,000 for males and females, respectively. For males, the highest DALYs in 2019 were observed in Hungary, Denmark, and Belgium for males (1009/100,000 and 818/100,000 and 798/100,000, respectively). For females, the highest DALYs in 2019 were observed in Denmark, the UK, and the Netherlands (742/100,000, 622/100,000 and 584/100,000, respectively). The lowest DALYs in 2019 in males were observed in France, Latvia, and Estonia (315/100,000, 343/100,000 and 344/100,000). For females, the lowest DALYs were observed in Latvia, Estonia, and Lithuania (140/100,000, 147/100,000 and 159/100,000, respectively). Between 2001 and 2019, the median change in DALYs for males was − 179/100,000, with a median percentage change of − 25.7%. For females, the median change in DALYs was − 11/100,000 with a median percentage change of − 4.8%. Over the observation period, 25 of 28 countries demonstrated a decrease in DALYs in males and 18 of 28 countries demonstrated a decrease in females.

### Joinpoint analysis for COPD prevalence

**Fig. 1:**
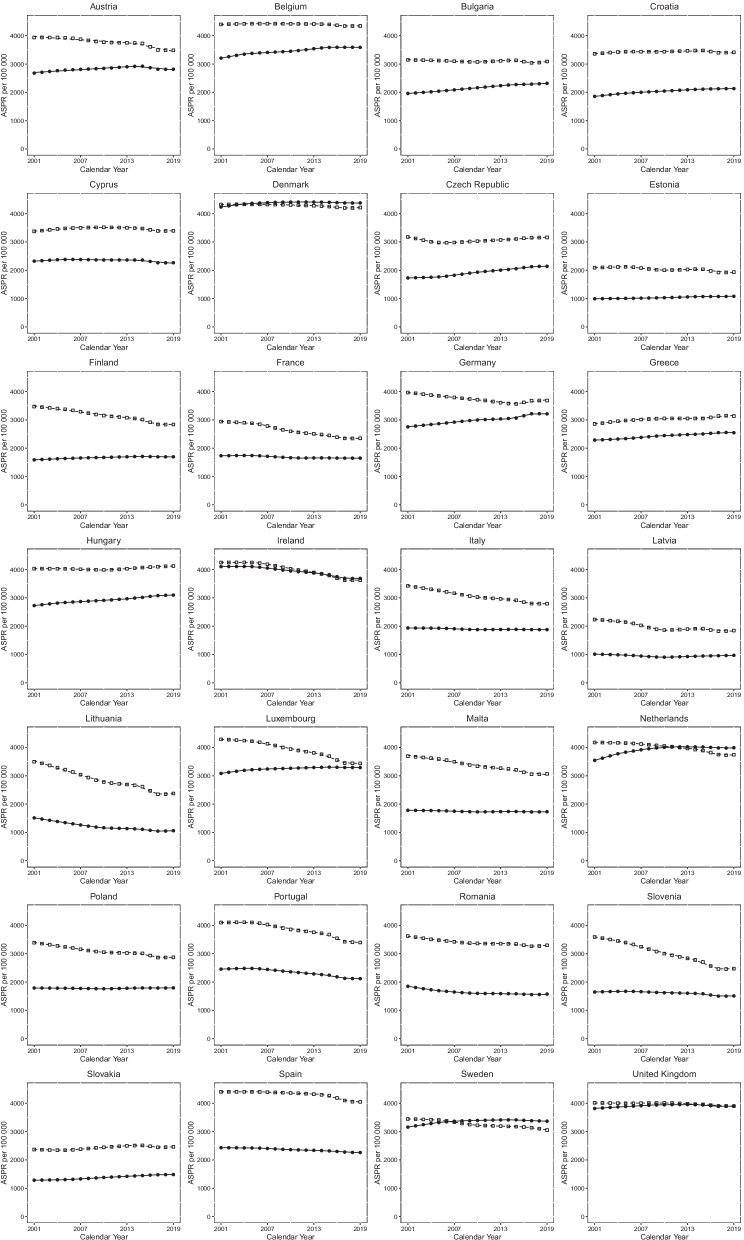
Prevalence trends for males and females in 28 European countries. Lines represent the results of Joinpoint analyses while symbols represent raw data, age-standardised prevalence rates (ASPR) per 100,000. Dashed and continuous lines represent males and females, respectively, while squares represent males and circles females

Sex-specific Joinpoint regression analyses for COPD ASPR between 2001 and 2019 are displayed in Fig. 1 and Additional file1: Table S1. Significant ASPR EAPCs for each trend are presented (*p-*value < 0.05). For most countries, trends in COPD ASPR were relatively flat. For males, 14 countries had at least one positive trend, seven countries had two positive trends, and only Greece demonstrated increases in all trends in ASPR. At the end of the observation period, COPD prevalence showed an increasing trend in 10 countries (Bulgaria, Cyprus, Czech Republic, Denmark, Germany, Greece, Hungary, Lithuania, Romania, and Slovakia). For females, 25 out of the 28 countries had at least one positive trend, 18 had at least two positive trends, 14 had at least three positive trends, and eight countries demonstrated increasing in all trends in ASPR (Bulgaria, Croatia, Czech Republic, Estonia, Germany, Greece, Hungary, and Slovakia). Ireland, Slovenia, and Spain demonstrated decreases in all trends. In addition to the eight countries with persistently increasing trends, four other countries showed increasing trends at the end of the observational period (Latvia, Lithuania, Poland, and the UK).

### Joinpoint analysis for COPD mortality

**Fig. 2:**
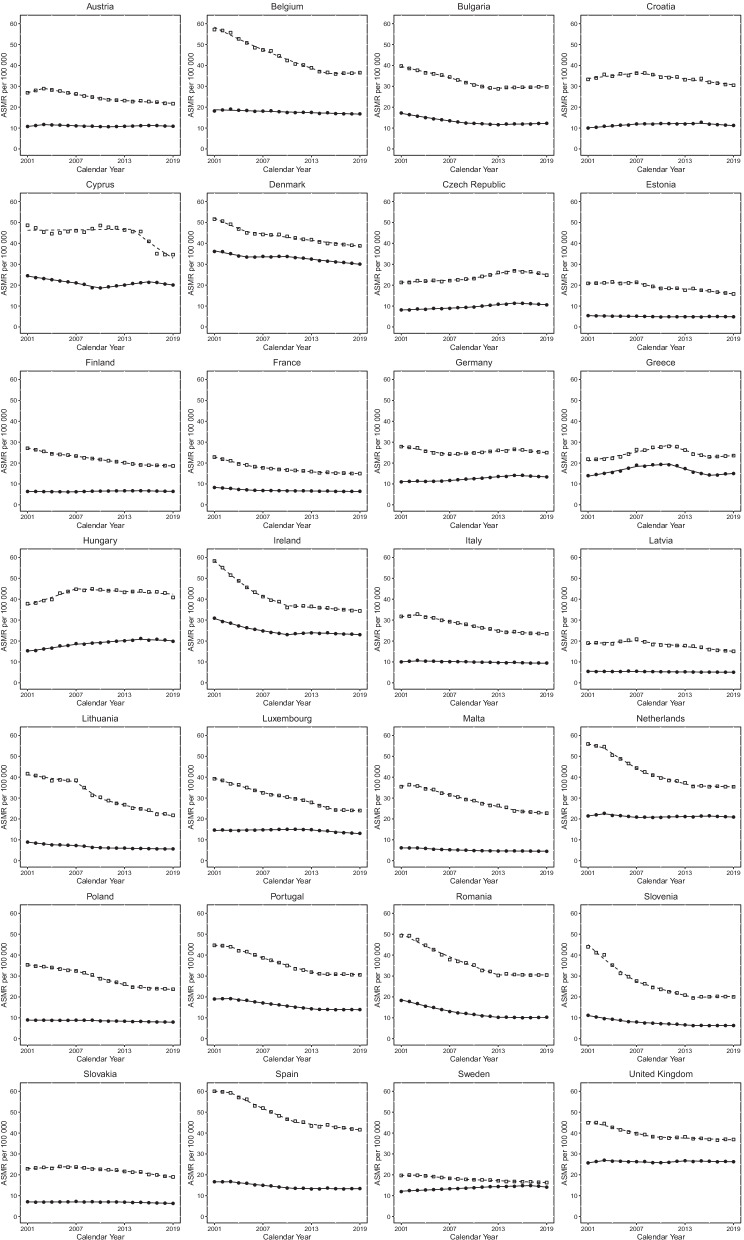
Mortality trends for males and females in 28 European countries. Lines represent the results of Joinpoint analyses while symbols represent raw data, age-standardised death rates (ASMR) per 100,000. Dashed and continuous lines represent males and females, respectively, while squares represent males and circles females

Sex-specific Joinpoint regression analyses for COPD ASMR between 2001 and 2019 are displayed in Fig. 2 and Additional file1: Table S1. Significant ASMR EAPCs for each trend are presented (*p-*value < 0.05). Most trends observed for both males and females show favourable decreases in COPD mortality rates across Europe, with a higher rate in males than females. Twelve countries for males and 8 for females showed negative trends throughout the period of observation. Other countries had variable positive and negative trends throughout. Four countries had a final positive trend for males (Belgium, Bulgaria, Greece, and Slovenia) and three for females (Bulgaria, Estonia, and Greece), representing increases in COPD mortality over the most recent periods assessed.

Joinpoint analyses of COPD DALYs and MIIs were also performed and can be found in the Additional file2: Figs. S1 and S2.

## Discussion

In this observational study of chronic obstructive pulmonary disease across the 28 European Union member states between 2001 and 2019, we have shown an overall decreasing prevalence of COPD for males. However, this decrease was not seen in some countries for males (5/28, 18% of countries), nor was it seen in most countries for females (17/28, 61% of countries). Further, some countries saw a higher prevalence in females than males by the end of the observation period. Although males have greater mortality than females, this gender-mortality gap decreased during the observation period, keeping with the changing burden of COPD prevalence. There has been a consistent decrease in mortality from COPD in both males (25/28, 89% of countries) and females (19/28, 68% of countries), as well as for disability-adjusted life-years in both males (25/28, 89% of countries) and females (18/28, 64% of countries). The ratio of COPD mortality-to-incidence demonstrated more favourable changes, with only two countries for males and three countries for females reporting an increase in the mortality-to-incidence index. Together, these findings are in contrast to previous reports, which had indicated that the burden of disease from COPD was reducing [1, 2]. Here we find these reductions may be plateauing and, in some cases, reversing.

COPD is of particular importance for policymakers, public health officials, and system-level healthcare as it represents the most prevalent chronic lung disease globally, and it is particularly amenable to interventions for prevention [4]. The principal objective of our investigation was to obtain current estimates of the burden of COPD across the EU. Furthermore, we sought to examine whether previously observed improvements in the burden of COPD have continued. Although a previous global analysis has assessed GBD data for COPD over a similar period, this is the first study to focus on Europe [1]. The 2015 Global Burden of Disease study reported an overall increase in global COPD mortality between 1990 and 2015 of 11.6%, however, when these data were controlled for an ageing population, the reported ASMR was significantly reduced by − 41.9% [1]. The greatest reductions in ASMR were seen in countries of high socioeconomic development, such as those in Europe. A previous report analysing COPD mortality in Europe between 1994 and 2010 demonstrated a linear decrease in mortality for males with minimal changes for females [2]; we observed similar mortality trends initially during the observation period. We also observed a higher relative reduction in ASMR in countries with higher ASMRs at the start of the observation period (2001). In more recent years, however, countries such as Austria, Belgium, Bulgaria, Denmark, Finland, France, Greece, Ireland, Italy, Luxembourg, Netherlands, Poland, Portugal, Romania, Slovenia, Spain, Sweden, and the UK—which had seen steep improvements in COPD mortality for males—have seen reductions in year-on-year mortality improvements and, in some cases, increases in mortality. Further, although prevalence has been decreasing in most countries for males, the prevalence of COPD in females is increasing in most countries.

This is not the first study to report an increasing prevalence in females in high-income countries. A previous report using the GBD identified an increase in COPD prevalence in females in several countries up to the year 2017 [4]. There is also a narrowing of the mortality gap between females and males. Potential explanations for this include increased longevity due to reduced mortality from illnesses such as cardiovascular disease and changing patterns in smoking behaviours amongst females [16]. The sex differences in COPD and the underlying factors contributing to this have been recently [16]. Hopefully, future population initiatives could also attempt to highlight these changing demographic trends.

Changes in COPD burden are unlikely to be the result of any one particular entity. They are more likely to result from a combination of factors that and due to the observational nature of this study, we are unable to infer causality. The observation period is of interest because, whilst globally tobacco use remains high, the majority of European countries have seen a fall in smoking rates, with data indicating that high levels of tobacco control correlate with higher quittance rates [17]. The relationship between air pollution and COPD is well documented but quantifying the precise attributable respiratory mortality from air pollution remains complex [18]. However, Europe is likely to have lower attributable mortality to air pollution than other parts of the world [19, 20]. Still, within Europe itself, the impact of pollution is variable and may contribute to the differences in disease burden observed between member states [21]. Observed trends may also be attributable, in part, to other factors such as improved knowledge and information with regard to exacerbating factors, treatment of respiratory and non-respiratory co-morbid conditions, and improvements in the diagnosis of chronic respiratory diseases. Indeed, during the early years of our observation period, a number of significant randomised controlled trials were published, generating and optimising the evidence base for pharmacotherapy in COPD; however, the latter years have seen few or no novel therapies [22]. COPD management has been advanced more recently through observational research to permit more accurate phenotyping of the population. Bundles of care have been developed using a combination of community interventions, including pulmonary rehabilitation and holistic assessment at hospital admission, with greater emphasis on smoking cessation [23, 24].

Compared with cardiovascular disease [25], the gains made in COPD mortality in Europe are relatively modest, and the failure to universally decrease COPD prevalence provide support for European initiatives such as the Chronic Airways Disease Early Stratification (CADSET) collaboration [26]. Such initiatives would help provide a framework for quality improvement in clinical care and outcomes, improve public engagement through knowledge sharing and public awareness, and enhance research and training and development in the field of chronic lung diseases. Future work may focus on inter-European public health policy on smoking cessation and air quality in addition to variation in COPD management.

The major strengths of this report are the total number of countries observed and the total duration of the observation period for analysis. We used standardised estimates of prevalence and mortality, which allows us to make comparisons between countries by removing the influence of country-specific demographics on these variables. To our knowledge, this is the first paper to provide a detailed European analysis of prevalence and mortality from COPD for the last 20 years. Despite this study’s strengths, our study carries a number of limitations—as with all studies using the GBD database—including specific limitations with case definitions of COPD [1]. The data that we report are observational with probable variations in diagnostic practices and medical provision between European countries. The prevalence estimates are particularly limited as countries are not required to maintain registries of disease incidence as is required for the cause of death. Therefore, any causal inferences about the impact of healthcare policies across Europe on COPD prevalence and mortality cannot be made. Additionally, the case definition of COPD is challenging, and there have been many iterations of the widely used Global Initiative for Chronic Lung Disease (GOLD) definitions of COPD. Notably, during the observation period for this study, there were revisions to the GOLD guidelines, including in 2012 and 2017, and it is possible, albeit unlikely, that these changing case definitions impacted upon recorded disease prevalence.

### Conclusion

While the burden of COPD remains high across European Union member states between 2001 and 2019, there have been overall reductions in mortality attributable to COPD. In contrast, while the prevalence of COPD continues to decrease in males across the majority of European nations, there are increasing trends in the prevalence of COPD in females. These data highlight the importance of continued efforts across the European Union to monitor and support initiatives to reduce the burden of respiratory diseases such as COPD.

## Supplementary Information

**Additional file1.** **Table S1**: Joinpoint analysis for male and female COPD prevalence from 2001 to 2019. APC indicates estimated annual percentage change. * Significantly different from 0 (P < 0.05). **Table S2**: Joinpoint analysis for male and female COPD mortality from 2001 to 2019. APC indicates estimated annual percentage change. * Significantly different from 0 (P < 0.05).

**Additional file2**. **Figure S1**: Mortality: Incidence ratio (MIR) trends for males and females in 28. **Figure S2**: DALY trends for males and females in 28 European countries Lines.

## Data Availability

All data was extracted from the publicly available Lancet Global Burden of Disease Database (https://www.healthdata.org/gbd/2019).

## Abbreviations

COPD: Chronic obstructive pulmonary disease
GBD: Global burden of disease
DALY: Disability adjusted life years
EU: European Union
WHO: World Health Organisation
GOLD: Global initiative for COPD
ASPR: Age standardised prevalence rate
ASMR: Age standardised mortality rate
ASIR: Age standardised incidence rates
MII: Mortality to incidence index
EAPC: Estimated annual percentage change
