# Supplementary material for: Trends in prevalence, mortality, and disability-adjusted life-years relating to chronic obstructive pulmonary disease in Europe: an observational study of the global burden of disease database, 2001–2019
Source: BMC Pulm Med. 2022 Jul 28;22:289. doi: 10.1186/s12890-022-02074-z (PMC9336030; doi:10.1186/s12890-022-02074-z)
Supplement: Supplementary file 1 — Additional file1. Table S1: Joinpoint analysis for male and female COPD prevalence from 2001 to 2019. APC indicates estimated annual percentage change. * Significantly different from 0 (P < 0.05). Table S2: Joinpoint analysis for male and female COPD mortality from 2001 to 2019. APC indicates estimated annual percentage change. * Significantly different from 0 (P < 0.05). [file 12890_2022_2074_MOESM1_ESM.docx]

**Supplementary table 1: Joinpoint analysis for male and female COPD prevalence from 2001 to 2019. APC indicates estimated annual percentage change. * Significantly different from 0 (P < 0.05)**

Male Prevalence:

| Country | Trend 1 | | Trend 2 | | Trend 3 | | Trend 4 | |
| --- | --- | --- | --- | --- | --- | --- | --- | --- |
|  | Years | APC | Years | APC | Years | APC | Years | APC |
| Austria | 2001 - 2014 | -0.48* | 2015 - 2019 | -1.55* | NA | NA | NA | NA |
| Belgium | 2001 - 2005 | 0.14* | 2006 - 2014 | -0.03* | 2015 - 2017 | -0.51* | 2018 - 2019 | -0.02 |
| Bulgaria | 2001 - 2010 | -0.30* | 2011 - 2014 | 0.54* | 2015 - 2017 | -0.96* | 2018 - 2019 | 0.58* |
| Croatia | 2001 - 2004 | 0.61* | 2005 - 2015 | 0.11* | 2016 - 2019 | -0.53* | NA | NA |
| Cyprus | 2001 - 2007 | 0.65* | 2008 - 2014 | -0.06 | 2015 - 2017 | -1.06* | 2018 - 2019 | 0.02 |
| Czech Republic | 2001 - 2005 | -1.78* | 2006 - 2019 | 0.49* | NA | NA | NA | NA |
| Denmark | 2001 - 2007 | 0.02 | 2008 - 2014 | -0.20* | 2015 - 2017 | -0.56* | 2018 - 2019 | 0.15 |
| Estonia | 2001 - 2005 | 0.44 | 2006 - 2010 | -1.25* | 2011 - 2014 | 0.48 | 2015 - 2019 | -1.30* |
| Finland | 2001 - 2014 | -1.05* | 2015 - 2017 | -2.20* | 2018 - 2019 | -0.26 | NA | NA |
| France | 2001 - 2006 | -0.62* | 2007 - 2009 | -2.53* | 2010 - 2017 | -1.40* | 2018 - 2019 | -0.34 |
| Germany | 2001 - 2015 | -0.75* | 2016 - 2019 | 0.98* | NA | NA | NA | NA |
| Greece | 2001 - 2007 | 0.94* | 2008 - 2015 | 0.15 | 2016 - 2019 | 0.77* | NA | NA |
| Hungary | 2001 - 2005 | -0.02 | 2006 - 2010 | -0.23* | 2011 - 2017 | 0.43* | 2018 - 2019 | 0.27* |
| Ireland | 2001 - 2006 | -0.11* | 2007 - 2014 | -1.19* | 2015 - 2017 | -1.96* | 2018 - 2019 | -0.03 |
| Italy | 2001 - 2010 | -1.40* | 2011 - 2014 | -0.67* | 2015 - 2017 | -1.67* | 2018 - 2019 | -0.16 |
| Latvia | 2001 - 2005 | -1.03* | 2006 - 2010 | -2.93* | 2011 - 2014 | 0.79 | 2015 - 2019 | -0.96* |
| Lithuania | 2001 - 2011 | -2.56* | 2012 - 2014 | -0.48 | 2015 - 2017 | -4.29* | 2018 - 2019 | 0.21 |
| Luxembourg | 2001 - 2005 | -0.37* | 2006 - 2014 | -1.29* | 2015 - 2017 | -2.75* | 2018 - 2019 | -0.34 |
| Malta | 2001 - 2019 | -1.13* | NA | NA | NA | NA | NA | NA |
| Netherlands | 2001 - 2006 | -0.16* | 2007 - 2014 | -0.63* | 2015 - 2017 | -1.69* | 2018 - 2019 | -0.16 |
| Poland | 2001 - 2010 | -1.21* | 2011 - 2014 | -0.09 | 2015 - 2017 | -1.73* | 2018 - 2019 | -0.14 |
| Portugal | 2001 - 2005 | -0.01 | 2006 - 2014 | -1.09* | 2015 - 2017 | -2.50* | 2018 - 2019 | -0.79 |
| Romania | 2001 - 2017 | -0.90* | 2010 - 2014 | -0.03 | 2018 - 2019 | 0.41 | NA | NA |
| Slovakia | 2001 - 2005 | -0.23* | 2006 - 2014 | 0.82* | 2015 - 2017 | -0.94* | 2018 - 2019 | 0.18 |
| Slovenia | 2001 - 2004 | -1.30* | 2005 - 2014 | -2.17* | 2015 - 2017 | -3.82* | 2018 - 2019 | -0.02 |
| Spain | 2001 - 2006 | -0.01 | 2007 - 2014 | -0.25* | 2015 - 2017 | -1.65* | 2018 - 2019 | -0.80* |
| Sweden | 2001 - 2016 | -0.26* | 2006 - 2010 | -1.16* | 2017 - 2019 | -1.13* | NA | NA |
| United Kingdom | 2001 - 2011 | 0 | 2012 - 2019 | -0.36* | NA | NA | NA | NA |

Female prevalence:

| Country | Trend 1 | | Trend 2 | | Trend 3 | | Trend 4 | |
| --- | --- | --- | --- | --- | --- | --- | --- | --- |
|  | Years | APC | Years | APC | Years | APC | Years | APC |
| Austria | 2001 - 2014 | 0.61* | 2015 - 2019 | -0.88* | NA | NA | NA | NA |
| Belgium | 2001 - 2004 | 1.52* | 2005 - 2010 | 0.47* | 2011 - 2015 | 0.79* | 2016 - 2019 | -0.02 |
| Bulgaria | 2001 - 2004 | 0.97* | 2005 - 2014 | 1.14* | 2015 - 2019 | 0.47* | NA | NA |
| Croatia | 2001 - 2004 | 1.55* | 2005 - 2007 | 0.97* | 2008 - 2015 | 0.69* | 2016 - 2019 | 0.20* |
| Cyprus | 2001 - 2005 | 0.63* | 2006 - 2014 | -0.11 | 2015 - 2019 | -0.96* | NA | NA |
| Czech Republic | 2001 - 2005 | 0.48* | 2006 - 2010 | 1.83* | 2011 - 2017 | 1.35* | 2018 - 2019 | 0.56 |
| Denmark | 2001 - 2004 | 0.85* | 2005 - 2008 | 0.32* | 2009 - 2012 | 0.07 | 2013 - 2019 | -0.13* |
| Estonia | 2001 - 2005 | 0.30* | 2006 - 2010 | 0.46* | 2011 - 2014 | 0.92* | 2015 - 2019 | 0.21* |
| Finland | 2001 - 2006 | 0.71* | 2007 - 2015 | 0.41* | 2016 - 2019 | -0.26* | NA | NA |
| France | 2001 - 2005 | 0.14* | 2006 - 2011 | -0.89* | 2012 - 2014 | 0.11 | 2015 - 2019 | -0.13* |
| Germany | 2001 - 2010 | 0.96* | 2011 - 2014 | 0.27* | 2015 - 2017 | 1.85* | 2018 - 2019 | 0.14 |
| Greece | 2001 - 2005 | 0.54* | 2006 - 2009 | 1.00* | 2010 - 2019 | 0.53* | NA | NA |
| Hungary | 2001 - 2004 | 1.10* | 2005 - 2013 | 0.56* | 2014 - 2017 | 0.97* | 2018 - 2019 | 0.30* |
| Ireland | 2001 - 2005 | -0.02 | 2006 - 2014 | -0.70* | 2015 - 2017 | -1.35* | 2018 - 2019 | -0.17 |
| Italy | 2001 - 2005 | -0.10* | 2006 - 2010 | -0.56* | 2011 - 2014 | 0.16* | 2015 - 2019 | -0.12* |
| Latvia | 2001 - 2005 | -0.87* | 2006 - 2010 | -1.66* | 2011 - 2014 | 1.06* | 2015 - 2019 | 0.67* |
| Lithuania | 2001 - 2010 | -2.96* | 2011 - 2014 | -0.58* | 2015 - 2017 | -2.50* | 2018 - 2019 | 0.53 |
| Luxembourg | 2001 - 2004 | 1.19* | 2005 - 2007 | 0.47* | 2008 - 2015 | 0.25* | 2016 - 2019 | -0.15* |
| Malta | 2001 - 2005 | -0.19 | 2006 - 2010 | -0.51* | 2011 - 2014 | 0.2 | 2015 - 2019 | -0.14 |
| Netherlands | 2001 - 2004 | 2.19* | 2005 - 2008 | 1.17* | 2009 - 2012 | 0.42* | 2013 - 2019 | -0.17* |
| Poland | 2001 - 2005 | -0.10* | 2006 - 2010 | -0.25* | 2011 - 2014 | 0.39* | 2015 - 2019 | 0.05 |
| Portugal | 2001 - 2005 | 0.31* | 2006 - 2014 | -1.03* | 2015 - 2017 | -1.96* | 2018 - 2019 | -0.48* |
| Romania | 2001 - 2005 | -2.17* | 2006 - 2009 | -1.29* | 2010 - 2019 | -0.28* | NA | NA |
| Slovakia | 2001 - 2005 | 0.36* | 2006 - 2011 | 1.15* | 2012 - 2017 | 0.95* | 2018 - 2019 | 0.26* |
| Slovenia | 2001 - 2005 | 0.35* | 2006 - 2014 | -0.51* | 2015 - 2017 | -1.89* | 2018 - 2019 | -0.07 |
| Spain | 2001 - 2006 | -0.13 | 2007 - 2010 | -0.58* | 2011 - 2014 | -0.35* | 2015 - 2019 | -0.65* |
| Sweden | 2001 - 2005 | 1.27* | 2006 - 2008 | 0.56* | 2009 - 2014 | 0.18* | 2015 - 2019 | -0.30* |
| United Kingdom | 2001 - 2009 | 0.40* | 2010 - 2014 | 0.05 | 2015 - 2017 | -0.52* | 2018 - 2019 | 0.04 |

**Supplementary table 2: Joinpoint analysis for male and female COPD mortality from 2001 to 2019. APC indicates estimated annual percentage change. * Significantly different from 0 (P < 0.05)**

Male mortality:

| Country | Trend 1 | | Trend 2 | | Trend 3 | | Trend 4 | |
| --- | --- | --- | --- | --- | --- | --- | --- | --- |
|  | Years | APC | Years | APC | Years | APC | Years | APC |
| Austria | 2001 - 2003 | 3.82* | 2004 - 2011 | -2.56* | 2012 - 2019 | -0.96* | NA | NA |
| Belgium | 2001 - 2015 | -3.37* | 2016 - 2019 | 0.21 | NA | NA | NA | NA |
| Bulgaria | 2001 - 2006 | -2.26* | 2007 - 2012 | -3.19* | 2013 - 2019 | 0.43* | NA | NA |
| Croatia | 2001 - 2007 | 1.32* | 2008 - 2019 | -1.42* | NA | NA | NA | NA |
| Cyprus | 2001 - 2014 | 0.08 | 2015 - 2019 | -6.76* | NA | NA | NA | NA |
| Czech Republic | 2001 - 2009 | 0.76* | 2010 - 2015 | 3.00* | 2016 - 2019 | -1.97* | NA | NA |
| Denmark | 2001 - 2005 | -3.28* | 2006 - 2019 | -1.17* | NA | NA | NA | NA |
| Estonia | 2001 - 2007 | 0.19 | 2008 - 2010 | -4.52* | 2011 - 2014 | -0.49 | 2015 - 2019 | -2.67* |
| Finland | 2001 - 2004 | -3.31* | 2005 - 2007 | -1.77* | 2008 - 2015 | -2.44* | 2016 - 2019 | -0.65* |
| France | 2001 - 2006 | -4.60* | 2007 - 2014 | -1.92* | 2015 - 2019 | -0.70* | NA | NA |
| Germany | 2001 - 2007 | -2.59* | 2008 - 2015 | 1.25* | 2016 - 2019 | -1.47* | NA | NA |
| Greece | 2001 - 2011 | 3.12* | 2012 - 2016 | -4.45* | 2017 - 2019 | 1.21 | NA | NA |
| Hungary | 2001 - 2007 | 3.22* | 2008 - 2019 | -0.48* | NA | NA | NA | NA |
| Ireland | 2001 - 2006 | -5.89* | 2007 - 2010 | -3.78* | 2011 - 2019 | -0.74* | NA | NA |
| Italy | 2001 - 2003 | 1.48 | 2004 - 2014 | -2.63* | 2015 - 2019 | -0.74* | NA | NA |
| Latvia | 2001 - 2007 | 1.16 | 2008 - 2019 | -2.37* | NA | NA | NA | NA |
| Lithuania | 2001 - 2007 | -1.43* | 2008 - 2010 | -7.69* | 2011 - 2019 | -3.56* | NA | NA |
| Luxembourg | 2001 - 2008 | -3.07* | 2009 - 2012 | -2.19* | 2013 - 2016 | -4.40* | 2017 - 2019 | -0.29 |
| Malta | 2001 - 2003 | 0.17 | 2004 - 2016 | -3.20* | 2017 - 2019 | -1.05 | NA | NA |
| Netherlands | 2001 - 2003 | -1.82 | 2004 - 2008 | -4.81* | 2009 - 2014 | -2.66* | 2015 - 2019 | -0.25 |
| Poland | 2001 - 2007 | -1.36* | 2008 - 2014 | -3.78* | 2015 - 2019 | -1.06* | NA | NA |
| Portugal | 2001 - 2003 | -0.87 | 2004 - 2014 | -3.17* | 2015 - 2019 | -0.13 | NA | NA |
| Romania | 2001 - 2013 | -4.07* | 2014 - 2019 | -0.01 | NA | NA | NA | NA |
| Slovakia | 2001 - 2006 | 0.79* | 2007 - 2015 | -1.37* | 2016 - 2019 | -2.78* | NA | NA |
| Slovenia | 2001 - 2007 | -7.99* | 2008 - 2014 | -4.50* | 2015 - 2019 | 0.45 | NA | NA |
| Spain | 2001 - 2003 | -0.6 | 2004 - 2011 | -3.34* | 2012 - 2019 | -1.06* | NA | NA |
| Sweden | 2001 - 2003 | 0.34 | 2004 - 2008 | -1.95* | 2009 - 2019 | -0.92* | NA | NA |
| United Kingdom | 2001 - 2009 | -2.22* | 2010 - 2019 | -0.37* | NA | NA | NA | NA |

Female mortality:

| Country | Trend 1 | | Trend 2 | | Trend 3 | | Trend 4 | |
| --- | --- | --- | --- | --- | --- | --- | --- | --- |
|  | Years | APC | Years | APC | Years | APC | Years | APC |
| Austria | 2001 - 2003 | 4.02* | 2004 - 2011 | -1.19* | 2012 - 2016 | 1.24* | 2017 - 2019 | -1.16* |
| Belgium | 2001 - 2019 | -0.65* |  |  |  |  |  |  |
| Bulgaria | 2001 - 2009 | -3.90* | 2010 - 2013 | -1.44* | 2014 - 2019 | 0.77* |  |  |
| Croatia | 2001 - 2007 | 2.75* | 2008 - 2015 | 0.37 | 2016 - 2019 | -2.33* |  |  |
| Cyprus | 2001 - 2007 | -2.43* | 2008 - 2010 | -4.01* | 2011 - 2016 | 2.58* | 2017 - 2019 | -2.49* |
| Czech Republic | 2001 - 2009 | 1.72* | 2010 - 2015 | 3.48* | 2016 - 2019 | -1.74* |  |  |
| Denmark | 2001 - 2005 | -2.10* | 2006 - 2010 | 0.13 | 2011 - 2019 | -1.28* |  |  |
| Estonia | 2001 - 2010 | -1.13* | 2011 - 2019 | 0.11 |  |  |  |  |
| Finland | 2001 - 2006 | -0.75* | 2007 - 2010 | 1.58* | 2011 - 2015 | 0.49* | 2016 - 2019 | -1.22* |
| France | 2001 - 2006 | -3.77* | 2007 - 2019 | -0.58* |  |  |  |  |
| Germany | 2001 - 2006 | 0.5 | 2007 - 2015 | 2.41* | 2016 - 2019 | -1.30* |  |  |
| Greece | 2001 - 2007 | 5.00* | 2008 - 2011 | 1.57 | 2012 - 2016 | -6.34* | 2017 - 2019 | 2.16 |
| Hungary | 2001 - 2007 | 3.32* | 2008 - 2015 | 1.53* | 2016 - 2019 | -0.87 |  |  |
| Ireland | 2001 - 2005 | -3.93* | 2006 - 2010 | -2.54* | 2011 - 2013 | 1.32 | 2014 - 2019 | -0.63* |
| Italy | 2001 - 2003 | 2.12 | 2004 - 2019 | -0.70* |  |  |  |  |
| Latvia | 2001 - 2019 | -0.48* |  |  |  |  |  |  |
| Lithuania | 2001 - 2004 | -4.94* | 2005 - 2007 | -1.52 | 2008 - 2010 | -5.44* | 2011 - 2019 | -1.07* |
| Luxembourg | 2001 - 2012 | 0.33* | 2013 - 2019 | -2.10* |  |  |  |  |
| Malta | 2001 - 2003 | -0.39 | 2004 - 2006 | -4.23* | 2007 - 2011 | -2.33* | 2012 - 2019 | -0.60* |
| Netherlands | 2001 - 2003 | 2.36* | 2004 - 2007 | -2.01* | 2008 - 2016 | 0.35* | 2017 - 2019 | -0.71 |
| Poland | 2001 - 2008 | -0.2 | 2009 - 2019 | -0.96* |  |  |  |  |
| Portugal | 2001 - 2003 | 0.58 | 2004 - 2014 | -2.89* | 2015 - 2019 | -0.02 |  |  |
| Romania | 2001 - 2008 | -5.62* | 2009 - 2013 | -3.83* | 2014 - 2019 | -0.15 |  |  |
| Slovakia | 2001 - 2012 | 0.04 | 2013 - 2019 | -1.52* |  |  |  |  |
| Slovenia | 2001 - 2006 | -5.87* | 2007 - 2014 | -3.09* | 2015 - 2019 | -0.34 |  |  |
| Spain | 2001 - 2003 | 0.05 | 2004 - 2011 | -2.68* | 2012 - 2019 | -0.07 |  |  |
| Sweden | 2001 - 2012 | 1.44* | 2013 - 2017 | 0.80* | 2018 - 2019 | -2.39* |  |  |
| United Kingdom | 2001 - 2003 | 2.13* | 2004 - 2010 | -0.58* | 2011 - 2013 | 1.12 | 2014 - 2019 | -0.25 |
