# Supplementary material for: Trends in prevalence, mortality, and disability-adjusted life-years relating to chronic obstructive pulmonary disease in Europe: an observational study of the global burden of disease database, 2001–2019
Source: BMC Pulm Med. 2022 Jul 28;22:289. doi: 10.1186/s12890-022-02074-z (PMC9336030; doi:10.1186/s12890-022-02074-z)
Supplement: Supplementary file 2 — Additional file2. Figure S1: Mortality: Incidence ratio (MIR) trends for males and females in 28. Figure S2: DALY trends for males and females in 28 European countries Lines. [file 12890_2022_2074_MOESM2_ESM.pdf]

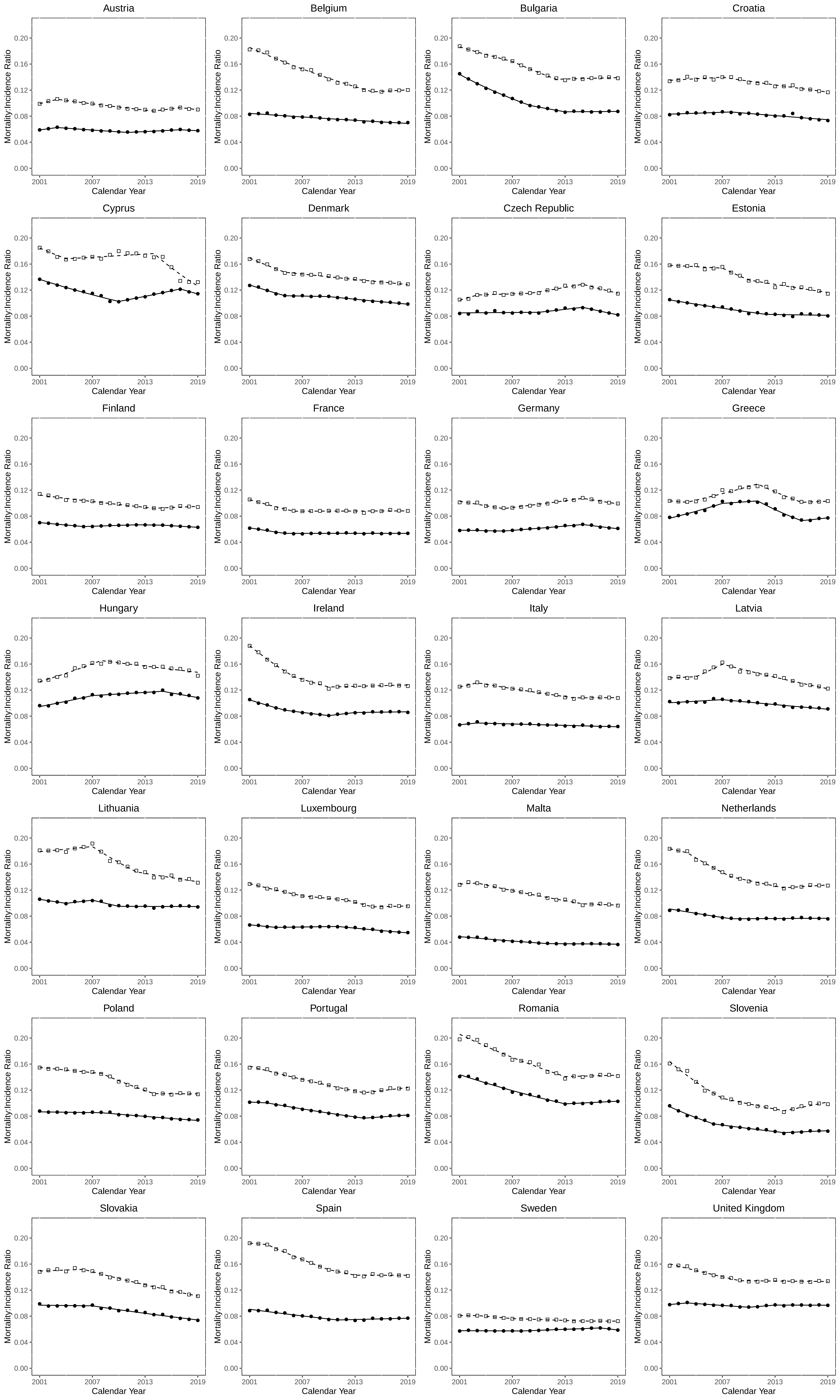
Austria
Belgium
Bulgaria
Croatia
Cyprus
Denmark
Czech Republic
Estonia
Finland
France
Germany
Greece
Hungary
Ireland
Italy
Latvia
Lithuania
Luxembourg
Malta
Netherlands
Poland
Portugal
Romania
Slovenia
Slovakia
Spain
Sweden
United Kingdom
Mortality:Incidence Ratio
Calendar Year
0.00
0.04
0.08
0.12
0.16
0.20
2001
2007
2013
2019

Supplementary figure 1. Mortality:Incidence ratio (MIR) trends for males and females in 28 European countries. Lines represent the results of Joinpoint analyses while symbols represent raw data, MIR. Dashed and continuous lines represent males and females, respectively, while squares represent males and circles females.

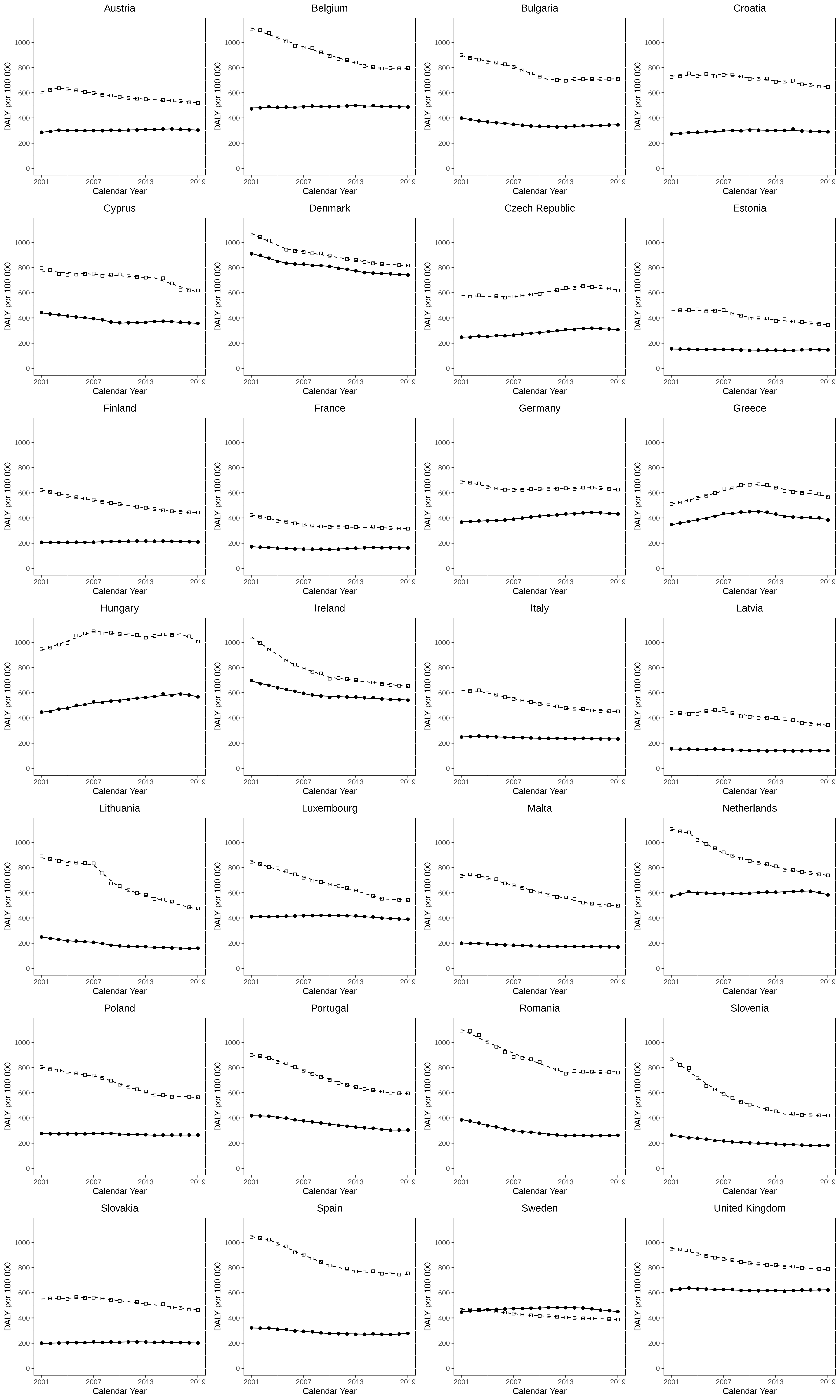
Austria
Belgium
Bulgaria
Croatia
Cyprus
Denmark
Czech Republic
Estonia
Finland
France
Germany
Greece
Hungary
Ireland
Italy
Latvia
Lithuania
Luxembourg
Malta
Netherlands
Poland
Portugal
Romania
Slovenia
Slovakia
Spain
Sweden
United Kingdom
DALY per 100 000
Calendar Year
0
200
400
600
800
1000
2001
2007
2013
2019

Supplementary figure 2. DALY trends for males and females in 28 European countries. Lines represent the results of Joinpoint analyses while symbols represent raw data, DALYs per 100,000. Dashed and continuous lines represent males and females, respectively, while squares represent males and circles females.
